# The oral-gut microbiome axis in inflammatory bowel disease: from inside to insight

**DOI:** 10.3389/fimmu.2024.1430001

**Published:** 2024-07-26

**Authors:** Aili Wang, Zihan Zhai, Yiyun Ding, Jingge Wei, Zhiqiang Wei, Hailong Cao

**Affiliations:** ^1^ Department of Gastroenterology and Hepatology, Tianjin Medical University General Hospital, Tianjin Medical University, Tianjin, China; ^2^ Department of Gastroenterology and Hepatology, Binzhou Medical University Hospital, Binzhou Medical University, Binzhou, Shandong, China; ^3^ Department of Orthodontics, Tianjin Stomatological Hospital School of Medicine, Nankai University, Tianjin, China; ^4^ Tianjin Key laboratory of Oral and Maxillofacial Function Reconstruction, Tianjin, China

**Keywords:** inflammatory bowel disease, ulcerative colitis, Crohn’s disease, oral microorganism, oral-gut microbiome axis

## Abstract

Inflammatory bowel disease (IBD) is an idiopathic and persistent inflammatory illness of the bowels, leading to a substantial burden on both society and patients due to its high incidence and recurrence. The pathogenesis of IBD is multifaceted, partly attributed to the imbalance of immune responses toward the gut microbiota. There is a correlation between the severity of the disease and the imbalance in the oral microbiota, which has been discovered in recent research highlighting the role of oral microbes in the development of IBD. In addition, various oral conditions, such as angular cheilitis and periodontitis, are common extraintestinal manifestations (EIMs) of IBD and are associated with the severity of colonic inflammation. However, it is still unclear exactly how the oral microbiota contributes to the pathogenesis of IBD. This review sheds light on the probable causal involvement of oral microbiota in intestinal inflammation by providing an overview of the evidence, developments, and future directions regarding the relationship between oral microbiota and IBD. Changes in the oral microbiota can serve as markers for IBD, aiding in early diagnosis and predicting disease progression. Promising advances in probiotic-mediated oral microbiome modification and antibiotic-targeted eradication of specific oral pathogens hold potential to prevent IBD recurrence.

## 1 Introduction

Inflammatory bowel disease (IBD) is a type of intestinal disease marked by chronic inflammation with an unclear cause, and it is most commonly associated with ulcerative colitis (UC) and Crohn’s disease (CD). Even though IBD can be diagnosed at any age, from early childhood to old age, most new cases are discovered in adolescence and the early stages of adulthood. It is often clinically manifested as diarrhea, abdominal pain, and even bloody stool, with a tendency to delay and relapse (1). The onset and duration of IBD vary, and the disease’s severity is connected to the scope and degree of the lesions. It is often accompanied by arthritis, iritis, skin lesions, oral ulcers, hepatobiliary diseases, osteoporosis, and other parenteral lesions (2). At present, the primary goals of IBD treatment are symptom relief and quality of life enhancement, but there is no cure (3).

The etiology and pathogenesis of IBD are uncertain, although multiple factors, including disrupted intestinal mucosal immune regulation, persistent intestinal infections, impaired intestinal barrier function, genetic predisposition, and environmental factors (4), are believed to have a role in the onset and development of the disease (5). Of note, substantial research efforts have been directed towards unraveling the connection between the gut microbiome and IBD (6). Numerous studies have demonstrated that patients with IBD have marked dysbiosis in their gut microbiome, including reduced bacterial diversity (7), instability within the bacterial community at inflamed mucosal sites (8), as well as increased bacterial translocation and overgrowth (9).

Oral manifestations are increasingly recognized as extraintestinal manifestations of IBD, including UC and CD. These manifestations can present as various oral lesions, such as aphthous ulcers, mucosal tags, angular cheilitis, gingival inflammation, and periodontal disease (2). Ingesting a significant quantity of disordered oral bacteria, a consequence of periodontitis, could potentially disrupt the balance of gut bacteria, thereby causing changes in bacterial metabolites, compromised gut barrier function, and immune dysfunction (10, 11). The prevalence and severity of these oral manifestations may correlate with the activity and extent of intestinal inflammation. These oral symptoms are crucial for professionals to be aware of since they could have a major influence on the quality of life of patients with IBD. The exact mechanisms underlying the oral manifestations of IBD are not entirely comprehended (12). Patients with IBD had higher levels of microorganisms linked to opportunistic infections than non-IBD patients (13). Furthermore, when compared to non-IBD controls, the gut microbiome of IBD patients is noticeably more comparable to the oral microbiome (14). However, it is believed that systemic inflammation, immune dysregulation, altered oral microbiota, and medication side effects may contribute to their development. Systemic inflammation associated with IBD can lead to increased production of pro-inflammatory cytokines (15), which may directly affect the oral mucosa (16). Immune dysregulation in IBD can result in an abnormal immune response to oral pathogens, leading to oral inflammation. Altered oral microbiota, characterized by an imbalance in the makeup of oral microbial communities, could also influence the pathogenesis of oral manifestations in IBD (17). The purpose of this review is to give a broad overview of the advancements made in the field of oral microbiology and its relationship with IBD.

## 2 Homeostasis of the oral microbiome

Extensive research has been conducted on the composition of the oral microbiome. More than 250 types of organisms from the oral cavity have been identified through *in vitro* isolation and characterization, including a number of key pathogens implicated in the development of dental cavities and periodontal disease, for instance *Tannerella forsythia*, *Streptococcus mutans*, *Aggregatibacter actinomycetemcomitans* and *Porphyromonas gingivalis* (*P. gingivalis*) (2). Over time, integrated approaches to understanding oral disease states from a multimicrobial perspective have emerged, attributing disease pathology to co-occurring microbial networks whose aggregate activity contributes to pathogenesis rather than just key pathogens. The constant exposure of oral microbial ecosystems to exogenous foreign substances determines the formation of microorganisms and their ability to survive in this environment, as well as the unique relationship between microbes and hosts that depends on factors of selection (18). The ability to select and bind tongue and cheek cells before teeth emerge and the capacity to compete with other microbial species are key traits of pioneering oral microbial colonists like *Streptococcus salivarius*, *Streptococcus mitis*, *Streptococcus gordonii*, and *Streptococcus sanguinis*. These traits make them ideal for this specific niche. The gut and the oral cavity are the beginning and the end of the alimentary tract’s microbial aggregation. Both include distinct microbiome linked to human health and illness (19), as well as some shared microbiota. The relationship between gut and mouth microbiota is intricate, erratic, and interwoven (20). They can maintain a precise balance under normal physiological settings, but an imbalance in crosstalk will lead to the onset and progression of illnesses (21). More and more evidence showed connections between the oral microbiome and digestive diseases like esophageal cancer, colorectal cancer, acute appendicitis (22), and IBD. The capacity of numerous oral bacteria to modify the inflammatory microenvironment and obstruct host signaling pathways that regulate cell survival, proliferation, and differentiation may provide a molecular explanation for this connection.

## 3 The concept of intestinal microbiota disturbance in IBD

There are more than 1,000 distinct types of bacteria in the human intestinal microbiome, primarily belonging to the *Bacteroidetes* and *Firmicutes phyla*, which contain both helpful and harmful microorganisms (23). A condition of homeostasis exists in the gut of healthy people because of ongoing interactions between the microbiome, the human body, and between components of the microbiome, which inhibit the overgrowth of pathogenic organisms (24). The microbiota and the human body are in a reciprocal relationship (25). Gut microbiome mimics an “organ” community that plays a vital role in our bodies, including the biological metabolism of bile acids, the synthesis and utilization of dietary compounds such as vitamins and amino acids, the synthesis of vitamins, increased immune levels, and the protection of intestinal function (26). In exchange, an environment rich in energy sources such as protein and carbohydrates enable gut microorganisms to flourish (27). In recent years, plenty of subjects have studied the significance of the gut microbiome in pathogenesis of IBD and its composition and metabolism (28).

However, changes in the composition of bacteria can disrupt the balance, leading to the proliferation of pathogens that inhibit the growth of beneficial members of the gut microbiome (29). This disruption makes the gut more vulnerable to various pathogenic hazards and has adverse effects on the host. This disruption of the microbiome ecosystem is referred to as an “ecological imbalance”. Ecological imbalance can be categorized into three distinct categories: reduced microbial diversity (30), depletion of symbiotic bacteria, overgrowth of opportunistic pathogens, infection with obligate intestinal pathogens and antibiotic therapy. In fact, ecological imbalance reflects shifts in the constitution of the microbiome, metabolic disturbances, and alterations in the distribution of bacteria that negatively impact the balance and can trigger tumorigenesis (31). One of the most susceptible sites for the extraintestinal symptoms of IBD is the mouth cavity, and multiple studies have revealed that IBD patients are more likely to develop periodontitis than non-IBD patients (32, 33). Furthermore, it was discovered that patients with IBD had more severe and more extensive periodontitis than patients with general periodontitis. Patients with IBD also had significantly higher mean probing depths, plaque and calculus indexes, sulcus hemorrhage indexes, and attachment loss (34). In addition, compared to healthy controls without periodontitis, patients with periodontitis have an increased chance of acquiring IBD (35).

## 4 Oral-gut microbiome axis

The oral-gut barrier, chemical separation, and physical barriers like bile and stomach acid keep the oral and intestinal microbiomes apart (36). Nevertheless, under certain circumstances, the integrity of the oral-intestinal barrier can be compromised, and acidity of stomach acid may decrease (37), allowing for the translocation and communication of microorganisms between these two organs. For instance, in newborns with immature barrier function, *Bifidobacterium* that colonize the intestines can migrate into the saliva (38).

The relationship between the oral and intestinal microbiomes exists even in individuals who are considered healthy (39). Research examining microbial strains present in fecal and saliva samples from 310 subjects in five different countries identified around 470 species of microorganisms (40). The study revealed that oral microorganisms can frequently travel from the mouth to the intestinum crassum and then colonize there in healthy individuals. Additionally, most oral microorganisms have the ability to be transferred to the gut (41). Ectopic colonization of oral microbiota in the healthy bowel could help maintain the physiological development of intestinal immunity. For example, intestinal colonization by the oral bacterium *Villanella*. may regulate host immunity (42). Oral microorganisms can be transferred to the gut through the blood as well as through the digestive tract.

### 4.1 Blood transmission route

Studies have demonstrated that mechanical actions like tooth brushing or dental procedures can result in the transmission of oral microorganisms to the blood through wounds (43). After oral bacteria enter the bloodstream, they usually colonize the joints, bones, liver, heart and other organs, resulting in disease (44). In addition, oral bacteria can invade dendritic cells and macrophages, colonize them, and then travel through the circulatory system to reach the highly vascularized intestinal tract (45), consequently spreading to the intestinal mucosa (46). However, there are also some oral microorganisms, such as *Fusobacterium nucleatum*, that express the Fap2 protein, which inhibits the activity of T cells, natural killer (NK) cells, and other immune cells by interacting with T cell immunoglobulin and ITIM domain (TIGIT) receptors on the surface of human immunocytes (47).

### 4.2 Digestive tract transmission route

**Figure 1:**
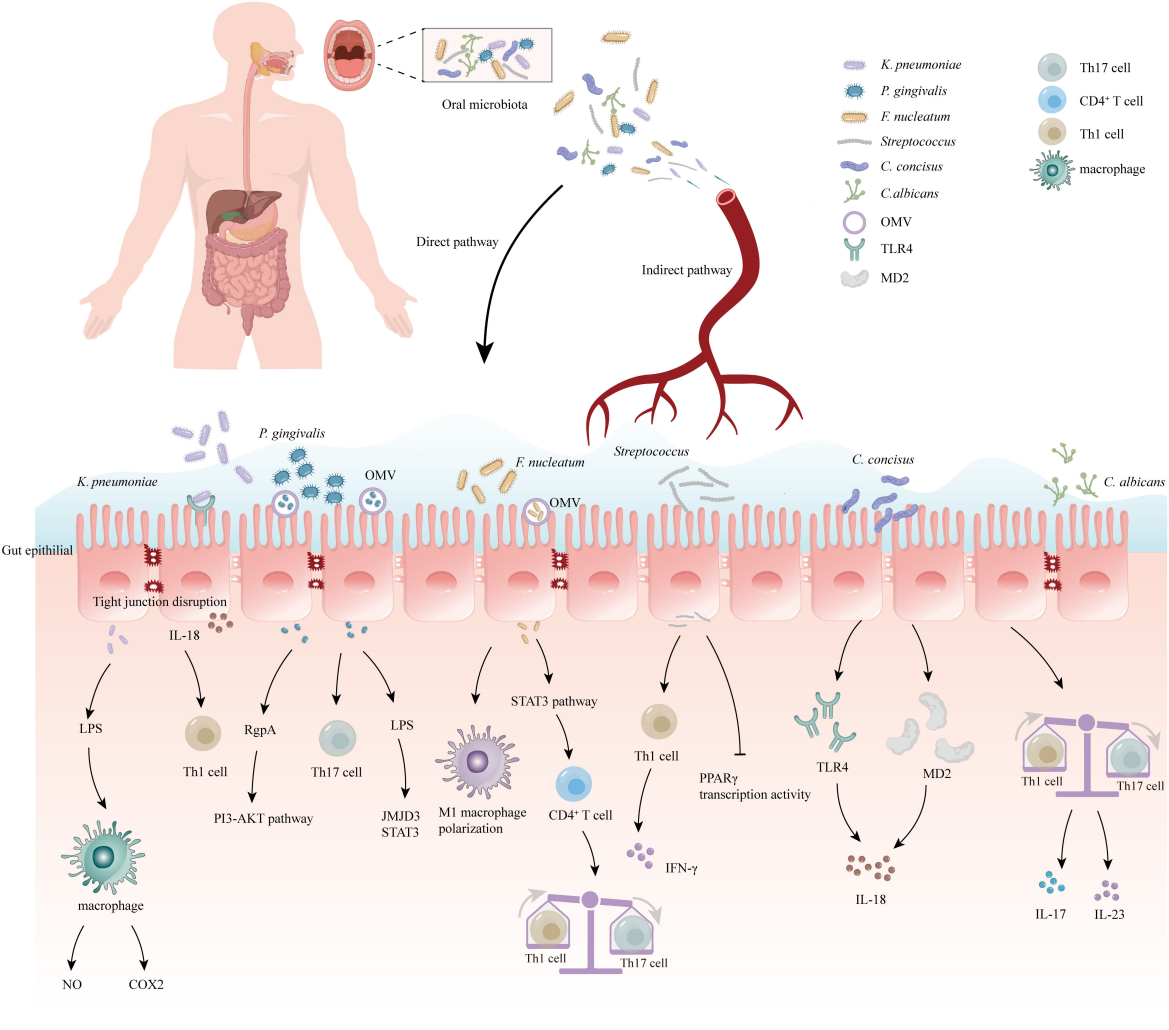
Correlation between oral microbiome and inflammatory bowel disease (IBD). The oral bacteria can induce the development of IBD via several mechanisms: (1) Destruction of the intestinal epithelial barrier: *P. gingivalis* and *K. pneumoniae* can downregulate the expressions of tjp-1 and occludin. (2) Release of pro-inflammatory cytokines: *F. nucleatum* and *K.pneumoniae* can induce stimulate the pro-inflammatory cytokine LPS. (3) Disruption of the host immune system and induction of immune escape: *F. nucleatum* and *Candida* imbalance the Th1/Th17 which induce inflammatory reactions. (4) Migration to the gut and activation of the inflammasome in colonic mononuclear phagocytes: K. pneumoniae and F. nucleatum can migrate to the gut and activate the inflammasome in immune cells, leading to intestinal inflammation. Outer-membrane vesicles (OMV), Toll-like receptor 4 (TLR4), myeloid differentiation protein-2 (MD2), Helper T 17 cell (Th17 cell), Cluster of differentiation 4+ T cell (CD4+ T cell), Helper T 1 cell (Th1 cell), Limited Power Supply (LPS), Nitric Oxide (NO), Phosphoinositide 3-Kinase (PI3K), Cyclooxygenase-2 (COX2), Interleukin-18 (IL-18), Interleukin-17 (IL-17), Interleukin-23 (IL-23), Jumonji domain-containing protein-3 (JMJD3), Signal transducer and activator of transcription (STAT3), Peroxisome proliferator-activated receptorγ (PPARγ), Interferon-γ (IFN-γ).

The relationship between the oral and intestinal microbiomes is facilitated by saliva, which serves as a medium for transporting various components (48). Saliva can carry effector cytokines and enzymes, both keratin-bound and free-floating bacteria, as well as functional inflammatory cell subpopulations like lymphocytes, neutrophils, and macrophages, to distant positions in the body (49). Additionally, saliva is a mixture of proteins, lipids, water, and mucins that make up mucus, which provides protection for these ingredients against the acidic environment of the stomach (50), allowing them to survive in the gastrointestinal tract (51). On average, an individual produces approximately 1 to 1.5 liters of saliva per day, which includes millions of microorganisms that can colonize the gut (52). In addition to the protective effect of saliva (53), there are also some oral pathogens with strong acid tolerance that can pass through the stomach, such as the well-known Streptococcus anginosus (54) (**Figure 1**).

## 5 Oral microbiome and development of IBD

Extensive research has provided evidence of the involvement of oral microorganisms in various extra-oral diseases, particularly those affecting the digestive system. The associations between these diseases and oral microorganisms are summarized in **Table 1**. It has been observed that oral microorganisms can reach the intestine through the ingestion of saliva, causing disruptions in the intestinal microecology and eventually contributing to the development of IBD. In the subsequent sections, we will delve into the detailed relationship between six specific types of microorganisms and IBD.

**Table 1:** Summary of various clinical experimental studies regarding the presence of oral bacteria in Inflammatory bowel disease (IBD).

Oral Associated Bacteria	Sampling/Size	Methods	Main Findings	References
*Fusobacterium nucleatum* *Streptococcus*	—Saliva and stool samples/ UC patients (*n*=42), CD patients (*n*=185), Controls (*n* =45)	16S rRNA gene sequencing	—Increased abundance of presented bacteria in IBD	Jin Imai et al. (14)
*Candida albicans*	—Feces, Oral swabs, colonic mucosa/UC patients (*n*=72), CD patients (*n*=18), Controls (*n*=36)	PCR-RAPD reaction	—Increased abundance of presented bacteria in IBD	Danuta Trojanowska et al. (55)
*Fusobacterium nucleatum*	—Colonic mucosa/ UC patients (*n*=4), CD patients (*n*=17), Controls (*n*=34)	qPCR	—Increased abundance of presented bacteria in IBD —high invasive Potential	Jaclyn Strauss (56)
*Campylobacter concisus*	—Saliva samples/UC patients (*n*=6),CD patients (*n*=13),Controls (*n*=23)	qPCR	—Increased abundance of presented bacteria in IBD	Fang Liu (57)
*Fusobacterium nucleatum*	—Feces samples/UC patients (*n*=20), CD patients (*n*=71), Controls (*n*=43)	qPCR	—Increased abundance of presented bacteria in IBD. —Correlated with patients’ disease activity	Hua Liu (58)
*Klebsiella pneumoniae*	—Feces samples/UC patients (*n*=51), CD patients (*n*=7), Controls (*n*=150)	16S rRNA gene sequences	—Increased abundance of presented bacteria in IBD and exacerbate intestinal diseases	Koji Atarashi (59)
*Fusobacterium,Haemophilus*, *Porphyromonas, Campylobacter*	—Feces samples/CD patients (*n*=43), Controls (*n*=18)	16S rRNA gene sequencing	—Increased abundance of presented bacteria in IBD	Kai Xia (60)
*Porphyromonas gingivalis*	—Feces samples/CD patients (*n*=11), Controls (*n*=8)	16S rRNA gene sequencing	—Increased abundance of presented bacteria in CD	Yu-Chen Lee (61)
*Streptococcus gallolyticus*	—Feces samples/IBD patients (*n*=44), Controls (*n*=40)	qPCR	—Increased abundance of presented bacteria in IBD	Ahmad Farajzadeh Sheikh (62)
*Campylobacter concisus*	—Feces samples/CD patients (*n*=54), Controls (*n*=33)	16S rRNA gene sequences,	—Increased abundance of presented bacteria in CD	Si Ming Man (63)
*Candida*	—Feces samples/ IBD patients (*n*=235), Controls (*n*=38)	16S rRNA gene sequences	—Increased abundance of presented bacteria in IBD	Harry Sokol (64)

Inflammatory bowel disease (IBD), Ulcerative colitis (UC), Crohn’s disease (CD), Polymerase Chain Reaction Random Amplified Polymorphic DNA reaction (PCR-RAPD reaction).

### 5.1 *P. gingivalis* and IBD

A well-known keystone pathogen for periodontal illnesses, *P. gingivalis* can spread from the mouth to the gut in mice, causing a dysbiosis of the gut microbiota characterized by an increased abundance of *Bacteroidetes* and a decreased abundance of *Firmicutes* (65). This weakens the function of the intestinal barrier by downregulating the expression of tjp-1 and occluding genes (66). *P. gingivalis* secretes gingipains that help them selectively inactivate pro-inflammatory substances generated by activated dendritic cells (DCs) and evade innate immune responses. In addition to the effects of metabolites, microbiome dysbiosis can produce abnormal immunological responses. Tim et al. showed that phagocytosis, NETosis, and CAMP activity are the three different ways that PPAD, which is the virulence factor of the oral pathogen *P. gingivalis*, defuses antibacterial neutrophil assaults (67). This indicates that PPAD plays a significant role in the escape of human natural immunity. Research by Potempa (68) and colleagues demonstrating PPAD-dependent complement system citrullination supports this theory.

Because *P. gingivalis* fimbriae facilitate bacterial adherence to and invasion of specific areas, they are essential for mediating the organism’s contact with host tissues (69). According to the findings from Lagha et al. (70), *P. gingivalis* penetrated and persisted in the gingival epithelial cells that were cultivated, and it caused the epithelial barrier to be damaged by breaking down the ZO-1 protein. The possible mechanisms are that the three ways that *P. gingivalis* reduced the amount of ZO-1 protein *in vivo* were via detaching intestinal mucus, invading epithelial cells of the intestine, and breaking down cytosolic ZO-1. Aleksandra et al. (71) showed that fimbriae isolated from the *P. gingivalis* mutant strain lacking in PPAD were unable to activate TLR2, suggesting that fimbriae constituents or other proteins related to fimbriae assembly must be citrullinated in order for TLR2 to activate on host cells.

Gingipains (Kgp, RgpA, and RgpB) are complex, lysine- or arginine-specific cysteine proteinases that are secreted to the microbes surface through a type IX secretion system (T9SS) (72) and released into the environment as cargo or as soluble proteins on outer membrane vesicles (OMVs). These proteinases have been identified as the primary pathogenic factors of *P. gingivalis*. When enzymatically inactivated, RgpA is the gingipain that mediates EGFR-mediated signaling. This is explained by the sequential motif that is specific to the hemagglutinin adhesion domain of RgpA and absent from Kgp (73). Izabela et al. showed that the PI3K-AKT pathway was activated by potent tyrosine residue phosphorylation caused by RgpA in the absence of enzymatic activity in EGFR. The pro-inflammatory response, endocytosis, differentiation, proliferation, and metabolism are all attributed to this route. Phosphorylation of T308 was the primary method by which inactive RgpA activated AKT, indicating the participation of the PDK1 protein, a phosphoinositide-1-dependent kinase (74). This stands in stark contrast to RgpA, which is active enzymatically, which reduces AKT phosphorylation and causes the kinase’s natural form to degrade (75). The mechanism of action of KYT-36 (76), a highly selective inhibitor peptide, against gingipain has been demonstrated in a recent study. This peptide offers potential as a therapeutic agent for the treatment of IBD and periodontitis in clinical settings.

Related studies have shown that the Th17 cell increase in periodontal tissues is stimulated by intestinal priming and oral *P. gingivalis* infection, which exacerbates periodontitis (77). It was observed that gut-translocated *P. gingivalis* can enter the Peyer’s patches (PPs) of the small intestine through microfold cells (M cells) in mice and induce a systemic response mediated by *P. gingivalis*-Th17 cells. However, when *P. gingivalis* was heat-inactivated and its proteins denatured, M cells were less likely to absorb it, resulting in reduced alveolar bone loss. This indicates that heat-inactivated denatured proteins of *P. gingivalis* were ineffective as components of the bacterial T cell antigen for uptake by M cells (78). In patients with periodontitis, an upregulation of CCL20, a chemokine involved in cell migration, has been observed in periodontal tissues (79). *P. gingivalis*-Th17 cell migration aggravates periodontitis progression, indicating that inhibiting the CCL20/CCR6 axis could prevent periodontitis from developing (80). To break the pathogenic gut-oral axis in the inflammatory oral disease process, one possible therapeutic target is to inhibit the invasion of pathobiont-responsive Th17 cells into the dental cavity.

Under normal conditions, the expression of JMJD3, a protein involved in epigenetic regulation, is low. However, different cellular stressors can cause JMJD3 to be expressed (81). Studies have shown that lipopolysaccharide (LPS) stimulation has the ability to activate JMJD3, which in turn controls genes linked to inflammation in peripheral macrophages (82). Along with increased levels of p-STAT3, STAT3, and RORγt, it was also found that *P. gingivalis*-LPS stimulated the expression of JMJD3 and further improved their interactions. This implies that JMJD3 might be targeting STAT3 and RORγt (83).

### 5.2 *Fusobacterium nucleatum* and IBD

The obligate anaerobic bacterium *F. nucleatum* is spindle-shaped, opportunistic, Gram-negative, and anaerobic (84). It is frequently found in the oral cavity, is a major factor in intrauterine infections linked to pregnancy complications such as stillbirth, neonatal sepsis, and preterm birth, and is present in the dental plague that precedes the beginning of periodontitis (85). The human intestine is not primarily colonized by *F. nucleatum*, which is actually a protobacterium, which can be both carcinogenic and antigenic. It is a very poor colonizing bacterium found in the gut microbiome of healthy humans, and most significantly, it has a distinct advantage in the microenvironment of colorectal tumors (86). Several symbiotic bacteria, in particular driver bacteria, are protobacteria in the human gut microbiome that are able to directly trigger damage to colon epithelial cells, thereby promoting the development of colorectal cancer. The gastrointestinal pathway is the main pathway for the colonization of oral *Fusobacterium* tuberculosis colorectal cancer tissue, and its virulent proteins FadA and Fap2 are mainly involved in its adhesion to colorectal cancer cells (87).

The mechanism of *F. nucleatum* in IBD has been thoroughly studied. *F. nucleatum* infection aggravates the inflammatory response and damages the integrity of the intestinal mucosal barrier (88). *F. nucleatum* produces a significant amount of hydrogen sulfide, a very toxic byproduct of cysteine metabolism that prevents colon cells from using butyrate effectively and causes persistent intestinal inflammation (89). According to certain research, the invasion and colonization of *F. nucleatum* may influence MUC2 production, which could lead to the development of intestinal inflammation (90, 91). Highly invasive *F. nucleatum* isolates from the lesions of CD patients showed considerably increased expressions of tumor necrosis factor alpha (TNF-α) and MUC2 compared with moderately invasive strains isolated from the healthy intestinal mucosa of control participants (92). By causing intestinal structure to be destroyed, upregulating the expression of TNF-α and IL-1β, and downregulating the expression of IL-10, *F. nucleatum* accelerates the development of DSS experimental colitis. F. Wei et al. suggest that *F. nucleatum*-EVs promote colitis by enhancing autophagy through the miR-574-5p/CARD3 axis (93).

The surface of *F. nucleatum* is home to a number of proteins, including RadD, FadA, Fap2, and FomA. These proteins not only facilitate its copolymerization with other bacteria but also aid in its attachment to host cells and trigger a number of immunological reactions in the host (94). *F. nucleatum* may cause colitis by controlling the skewing of M1 macrophages (95). Liu et al. found that by increasing the release of cytokines including IL-1β, IL-6, and IL-17 and by triggering the STAT3 signaling pathway, *F. nucleatum* may worsen intestinal inflammation by promoting the growth of CD4^+^ T cells and their differentiation into Th1 and Th17 cells (58). Li et al. found that patients with UC had higher levels of FadA genes and *F. nucleatum*, particularly those with severe pancolitis, indicating a potential role for FadA in the pathophysiology of UC (96). However, further research is required to clarify the precise mechanism of this connection and the functions of FadA genes and *F. nucleatum* in UC. Yan et al. found that non-surgical periodontal therapy triggers intestinal microbiome regulation and promotes a rehabilitative and healthy microbial environment by using the ApoE^−/−^ mice model (97). Furthermore, studies showed that *F. nucleatum* could interact with many other microorganisms and showed a synergistic effect of virulence when it was co-infected with other pathogens (98). A down-regulation of the mean relative abundance of probiotics, like *Clostridium prai*, coincides with an increase in the abundance of opportunistic pathogens, including *F. nucleatum* (99). The culture supernatant of *F. nucleatum* has an obvious inhibitory effect on *Clostridium prai* and *Bifidobacterium lactis* and can reduce the activity of *Lactobacillus rhamnosus* in high concentration. *F. nucleatum* can improve the efficiency of *E. coli* penetrating vascular endothelial cells (100). In addition, Duan et al. (101) found that *Lactobacillus rhamnosus* could significantly improve the damage to intestinal epithelial cells caused by infection with *F. nucleatum*. A variety of probiotics have been proven to help maintain intestinal barrier integrity and relieve or prevent DSS-induced intestinal inflammation in mice with UC, and *F. nucleatum* may further affect the recurrence of IBD through interaction with these probiotics (102). All the above studies indicated that *F. nucleatum* could jointly affect the occurrence and development of IBD through interactions with other microorganisms, but the specific effects and mechanisms remain unclear.

### 5.3 *Streptococcus gallolyticus* and IBD

Nearly every area of the human body has *Streptococcus*, which is the predominant species in the mouth (103). *Streptococcus* is divided into eight different groups, including *pyogenic, mitis, bovis, sanguinis, anginosus, downei, mutans, and salivarius* (104). At present, the oral cavity contains all groups, with the exception of the pyogenic and *bovis* groups. The largest group found in the mouth is the *mitis* group, with 20 species (105). Some researchers have reported increased amounts of *Streptococcus* in the intestines of patients with irritable bowel syndrome (IBS). These *Streptococcus* bacteria probably translocated from the mouth (106).

In subsequent investigations, studies found that oral *Streptococcus* can indeed migrate from the mouth and colonize the gut, even in healthy people (107). In a recent study, patients with IBD had higher concentrations of *Streptococcus* in their intestines (108). And the researchers confirmed that the *Streptococcus* originated in the mouth. Therefore, oral streptococci are the only shared genus (109). *Streptococcus sanguinis* ATCC10556 and TW289 from oral *Streptococcus* have been shown to aggravate colitis in mice by infecting liver cells and causing interferon gamma secretion, which leads to colitis aggravation. The specific mechanism may be that after infection with *Streptococcus* TW289 and ATCC10556, the Th1 immune response in the blood is activated, resulting in increased secretion of IFN-γ by T cells in the blood and spleen, which promotes the aggravation of inflammation (110).

In addition, *Streptococcus* mutans serotype k strain TW295 also has the effect of aggravating colitis. It infects the liver through the blood system and increases the expression of IFN-γ in the liver, thus aggravating colon inflammation (111). While TW295 is difficult to reach the colon through the complex physiological environment of the digestive tract, which is primarily brought on by blood dental surgery (112). However, not all oral *streptococci* are detrimental. One of the earliest bacteria to colonize the human mouth cavity and digestive system after birth is *Streptococcus salivarius*, which may be important for the development of immunological homeostasis (113). In mouse models, *Streptococcus salivarius* JIM8772 strains showed significant palliative effects on colitis. It was found that live *Streptococcus salivary* strains down-regulated the pro-inflammatory chemokine interleukin-8 (IL-8) secreted by intestinal epithelial cells *in vitro* and prevented the activation of the nuclear transcription factor-κB (NF-κB) pathway (114). Commensal *Streptococcus salivarius* can also regulate PPARγ transcription activity in human intestinal epithelial cells and significantly reduce the expression levels of I-FABP and Angptl4, thus contributing to the preservation of the health and equilibrium of the host environment (115).

### 5.4 *Campylobacter concisus* and IBD

A single polar flagellum, located at either or both sides of the bacterium, enables the majority of gram-negative, spiral- or curved-shaped *Campylobacter* species to move in a manner akin to a corkscrew (116). In the digestive tracts of different animals, the majority of *Campylobacter* bacteria exist as regular microbiomes. *C. concisus* is regarded as a pathogen for intestinal and oral disorders and is found in the digestive tract and oral cavity of IBD patients (117). PCR detection showed that the detection rate of *C. concisus* in the saliva of patients with UC was 100%, 85% in patients with CD, and only 75% in healthy individuals. *Campylobacter* has been presented in many studies to raise the chance of IBD (118). Compared with healthy controls, the incidence of *Campylobacter* in intestinal biopsies and stool samples collected from patients with IBD significantly increases. Especially *C. concisus* is more likely to colonize the proximal colon. The majority of *Campylobacter* species are oral bacteria found in humans rather than zoonotic species (119). The innate environmental factor for *C. concisus* colonization in the intestine is hydrogen gas. H_2_ gas significantly affects *C. concisus* growth. In laboratory culture, *C. concisus* grows very slowly in anaerobic settings but not at all in microaerobic conditions. H_2_ makes it possible for *C. concisus* to grow in microaerophilic environments and greatly accelerates its growth in anaerobic environments. The human intestine has microaerobic to anaerobic atmospheric conditions, with hydrogen, carbon dioxide, methane, and other gases making up the majority of colonic gases. In view of this, *C. concisus* may have the ability to offer a constant amount of available H_2_ for growth in their intestinal environment (120). Although the relationship between oral *Campylobacter* and IBD has been proven, the specific mechanism is still not very clear. Some oral *C. concisus* strains have owned the zonula occludens toxin (Zot) gene from viruses (pre-phages), and *C. concisus* zot has the same conserved base sequence as the human zonulin receptor binding domain and the cholera vibrio Zot receptor binding domain. The mechanism by which Zot-positive strains of *C. concisus* may promote the start and recurrence of IBD is primary barrier dysfunction, and *Campylobacter* can also activate inflammasome signaling in macrophages through Zot, thereby increasing the expression levels of TNF-α and IL-8 (121, 122). *C. concisus* isolated from the mouth and small intestine of IBD patients is highly invasive to HT-29/Caco2 cell lines, can upregulate the expression of MD-2 and TLR4 on cell surfaces, induce IL-8 production, increase intestinal epithelial permeability, and promote cell apoptosis. This suggests that *Campylobacter* may repeatedly enter the digestive tract with saliva and colonize the intestine, causing IBD in susceptible populations (123–126).

### 5.5 *Klebsiella pneumoniae* and IBD

A German microbiologist named Edwin Klebs made the initial discovery of *K. pneumoniae* (127). It is a widespread environmental bacterium that can be found on plants, in sewage, surface water, and soil. It belongs to the *enterobacteriaceae* family and is encapsulated, rod-shaped, gram-negative, and non-motile (128). Moreover, the bacteria are also known to colonize animal mucosa, including those of the gastrointestinal system and oral cavity. However, studies have shown significant differences between the *Klebsiella* genus identified in healthy human saliva and *K. pneumoniae* isolated in the saliva of patients with IBD (59, 129, 130). What’s more, recent research has discovered that the ectopic colonization of oral *Klebsiella* genus can lead to the activation of inflammasomes in resident macrophages, thereby exacerbating colitis (131). Studies have proved that when *K. pneumoniae* was taken orally, NF-κB activation in the colon, lipid peroxidation, and production of TNF-α, COX-2, IL-1β, and ZO-1 increased, whereas tight junction-associated proteins claudin-1, ZO-1, and occludin decreased (132). In animals with TNBS-induced colitis, *K. pneumoniae* also worsened the expression of tight junction-associated proteins and inflammatory markers (133). *K. pneumoniae* generated lipopolysaccharide and β-glucuronidase, which effectively stimulated the production of NO and COX-2 in mice peritoneal macrophages (134). From the colon tissues of colitis patients, they recovered *K. pneumoniae J* (KLPJ), a strain of KLP. This bacterial strain has the ability to cause and develop colitis, mediated by dextran sodium sulfate. It can also activate caspase-11 inflammasomes and cause mature IL-18 to be produced in colon epithelial cells and intestinal organoids (135). *K. pneumoniae* isolated from the salivary microbiome of 2 CD patients, specifically Kp-2H7, can activate epithelial cells and DCs through the TLR4 signaling pathway, stimulate IL-18 secretion, and cause recruitment and activation of Th1 cells, thereby leading to colitis (59). *K. pneumoniae* migrates from the mouth through the digestive tract and colonizes the intestines under harsh conditions. Through fermentation, *K. pneumoniae* generates hydrogen (H_2_) to counteract the damaging effects of NO and ROS. These properties keep it active in the mouth. In addition, exopolysaccharide capsules are its most important structure. By blocking immune cell phagocytosis, innate immune response activation, and complement activation-induced lysis avoidance, the capsule provides protection against the host immunological response (136, 137).

### 5.6 *Candida albicans* and IBD

Fungi actively participate in affecting health and disease in the intricate and multidimensional relationship that exists between the digestive system and the resident fungal community (the fungal community). *Candida* is the predominant fungal genus in the digestive tract and mouth of healthy individuals (138). *Candida* is suitable for colonization in various places in the digestive tract and mouth, increasing the possibility that *Candida* from the mouth will migrate to the intestine to cause disease. In a study of *Candida* colonization in the digestive tracts of patients undergoing hematopoietic stem cell transplantation (HSCT), the same candida genotypes were found in oral and intestinal samples. Oral *Candida* colonization is a risk factor for intestinal *Candida* colonization (139). In addition, *Candida* is closely associated with the development of IBD. Research has shown that the abundance of *C. albicans* in IBD patients, especially those with sudden inflammation, increases significantly. They verified a noteworthy rise in the abundance of *C. albicans* in IBD patients, especially during flare-ups. In mouse models, it was discovered that *Candida* tube feeding exacerbated the DSS model without impacting weight loss or diarrhea. This was evidenced by increased intestinal leakage (FITC-glucan assay, serum BG, endotoxemia, and blood bacterial load), a more severe colon histology, and higher mortality. Higher levels of pro-inflammatory cytokines are induced in intestinal and blood tissues by DSS^+^ *Candida* (140). And it has been found that colitis can be alleviated by preventing the propagation, pathogenicity, and colonization of *Candida* (141). Compared to healthy individuals, *C. albicans* colonized CD patients and their primary healthy relatives (HRs) more frequently and more severely. There was a correlation found between the colonization of *C. albicans* in the HRs and the antibodies against Saccharomyces cerevisiae antibodies (ASCAs) found in the sera of patients with CD (142). In addition, ASCA is a recognized marker for the clinical distinction between CD and UC (143). CD is associated with genes that are involved in the *Candida* glycogen response. For nucleotide-binding oligomerization domain-containing proteins (NOD) 1 and NOD2, as well as the NOD-like receptor family (133), genes encoding cytokines or their receptors, like IL-23 or IL-17, and pyrin domain-containing 3 (NLRP3), the direct impact on inflammatory responses is particularly evident. Autophagy is a significant mechanism in the development of IBD. By negatively regulating adaptive immunity and the activation of T cells, it makes patients susceptible to infection by *C. albicans* (144). Research has demonstrated that in patients with UC, severe cases are associated with the presence of “highly invasive” *Candida* strains, which can produce *Candida* to damage macrophages and promote the pro-inflammatory factor (145).

## 6 Conclusion and future prospects

There is increasing evidence in favor of a reciprocal relationship between IBD and the oral microbiota. The oral microbiota is one of the important symbiotic partners in the mouth and the human body. However, periodontal disease can cause systemic disease through the spread of inflammatory mediators and bacterial components through the blood. Current research has found that oral periodontal bacteria passing through the digestive tract can change the intestinal microbiota and cause ectopic colonization of the intestine, thereby exacerbating intestinal inflammation (146). We emphasize the significance of several oral bacteria in IBD via the oral-intestinal axis in this review. These include *P. gingivalis*, *F. nucleatum*, *C. concisus*, *K. pneumoniae*, and *C. albicans*.

Microbiota research on IBD has primarily focused on the gut microbiota, but emerging evidence suggests a potential link between dysbiosis of the oral microbiota and the development of IBD. The oral microbiota may have an impact on the inflammatory response of IBD, leading to worsening of the condition in IBD patients with periodontitis (147). Significant dysbiosis in the oral microbiota of IBD patients has been observed, indicating a potential role of the oral microbiota in the pathogenesis of the disease (148). Firstly, alteration in the oral microbiota can serve as markers for IBD, aiding in early diagnosis and predicting disease progression. Some studies have already identified variations in the composition of the oral microbiota between healthy people and IBD patients, suggesting that oral microbiota analysis could be used as an adjunct diagnostic tool for IBD (149, 150). Secondly, the oral microbiota can be a therapeutic target, and modulating the oral microbiota may offer a new approach for treating IBD, for example, reducing the abundance of oral bacteria in the intestine by using mouthwash (151). Future advancements in targeting probiotics for the oral microbiota and eliminating specific oral pathogens with targeted antibiotics hold promise for preventing IBD recurrence. However, this topic of study is still in its infancy, so further research is required. In the future, combining the investigation of oral microbiota with the monitoring of particular microbial products from the oral to the gut may provide fascinating new insights into the oral-gut microbiome axis.

## Author contributions

AW: Conceptualization, Data curation, Formal analysis, Funding acquisition, Investigation, Methodology, Project administration, Resources, Software, Supervision, Validation, Visualization, Writing – original draft, Writing – review & editing. ZZ: Conceptualization, Data curation, Formal analysis, Funding acquisition, Investigation, Methodology, Project administration, Resources, Software, Supervision, Validation, Visualization, Writing – original draft, Writing – review & editing. YD: Conceptualization, Data curation, Formal analysis, Funding acquisition, Investigation, Methodology, Project administration, Resources, Software, Supervision, Validation, Visualization, Writing – original draft, Writing – review & editing. JW: Conceptualization, Software, Supervision, Visualization, Writing – review & editing. ZW: Writing – review & editing. HC: Conceptualization, Data curation, Formal analysis, Funding acquisition, Investigation, Methodology, Project administration, Resources, Software, Supervision, Validation, Visualization, Writing – review & editing.
